# Transcatheter closure of a ruptured sinus of valsalva: a systematic review of the literature

**DOI:** 10.3389/fcvm.2023.1227761

**Published:** 2023-08-25

**Authors:** Aryan Ayati, Neda Toofaninejad, Ali Hosseinsabet, Fatemeh Mohammadi, Kaveh Hosseini

**Affiliations:** Cardiology Department, Tehran Heart Center, Tehran University of Medical Sciences, Tehran, Iran

**Keywords:** sinus of valsalva, percutaneous closure, ruptured sinus of valsalva aneurysm, transcatheter, aneurysm sinus of valsalva

## Abstract

**Background:**

Ruptured sinus of Valsalva (RSOV) is a rare pathology, and current data regarding its symptoms, anatomy, associated pathologies, and appropriate therapeutic approaches are scarce. Transcatheter closure (TCC) has been performed in multiple cases; however, the information on its success rate and complications is limited.

**Methods:**

Two independent reviewers performed an advanced search based on inclusion criteria on Scopus, PubMed, and Embase from January 1985 through July 2022. The main search terms were “Sinus of Valsalva”, “Rupture/Aneurysm”, and “Transcatheter/Catheter/Device”.

**Results:**

Totally, 1,017 relevant articles from the 3 databases were retrieved. After the final review and appraisal, 94 articles describing 407 patients who underwent the TCC of RSOV were included. Males comprised 65% of the studied patients, and the average age was 34.5 years. The total success rate of TCC was 95.6%. Forty-nine patients (12%) developed complications, the most significant of which were sustained residual shunts in 7 patients (1.7%), substantial new onset or progression of aortic insufficiency in 6 (1.5%), and RSOV recurrence in 6 (1.5%). Post-interventional mortality was reported in only 2 patients (0.5%).

**Conclusions:**

The present study is the first systematic review of available data regarding the TCC of RSOV principally comprising case series and case reports. Although TCC seems a good option, precise patient selection is mandatory.

## Introduction

Sinus of Valsalva aneurysm (SOVA) is an uncommon heart defect with an estimated prevalence of 0.2%–0.9% in cardiac surgery patients (1). SOVA can be divided into congenital and acquired categories based on its etiology. Congenital SOVA, which is more prevalent in male Asians (2), arises from the aberrant development of the bulbus cordis (3). Accompanying anomalies, such as ventricular septal defect, aortic insufficiency, and bicuspid aortic valve, are also frequently detected in congenital SOVA. Acquired SOVA can be associated with previous surgeries, atherosclerosis, endocarditis, syphilis, and other injuries (3, 4).

High-pressure flow in the proximal aorta, accompanied by the congenital or acquired weakness of the aortic wall, can form SOVA. The aneurysmal sinus can then rupture into an adjacent heart chamber, creating ruptured sinus of Valsalva (RSOV). The right, left, and non-coronary sinuses are, respectively, adjacent to the interventricular septum, the left ventricular free wall, and the interatrial septum (5). These proximities can explain the detection of each rupture root based on the SOVA origin.

SOVA predominantly occurs in the right coronary sinus (70%). SOVA usually remains asymptomatic prior to a rupture into adjacent structures. The rupture results in communication between the aorta and a heart chamber, leading to progressive heart failure (6). If left untreated, patients with RSOV have poor prognoses and high mortality rates, necessitating prompt interventions to close the ruptured fistula (7, 8).

The standard treatment for RSOV has been surgical repair. Nonetheless, increasing evidence suggests that the transcatheter closure (TCC) of RSOV is a viable, less invasive alternative (9). Studies regarding the TCC of RSOV are limited to case reports and case series, and large-scale clinical trials have yet to be performed (6). Conducting a systematic review of the existing evidence on the TCC of RSOV is crucial to attaining a comprehensive understanding of this treatment, and it should encompass patient characteristics, anatomic features of the lesion, types of devices employed, and potential complications. Such knowledge can enhance patient selection and confer deeper insights into potential outcomes.

The present study is a review of the current literature on the TCC of RSOV through an assessment of a summary of all relevant case series and case reports.

## Methods

### Search profile

This systematic review was conducted according to the latest Preferred Reporting Items for Systematic Reviews and Meta-Analyses (PRISMA) guidelines (10). On July 12, 2022, we performed a broad systematic search in the Scopus, PubMed, and Embase electronic databases. The main search terms were “Sinus of Valsalva”, “Rupture/Aneurysm”, and “Transcatheter/Catheter/Device”. The search syntax of each database is reported in Supplementary Table S1. Our search was limited to articles in the English language. All clinical studies, composed of original articles, case series, case reports, and letters to the editor, were retrieved. Additionally, references were searched for further related studies.

### Study selection

The investigations selected were clinical studies on pediatric or adult patients with RSOV or SOVA and case reports or case series with TCC as the treatment strategy.

The investigations excluded were animal studies, review articles, commentaries, editorials, and conference articles. Also excluded were studies with overlapping patients. (Newer investigations with more patients were included in this case, and previous studies were excluded.) Additionally, studies where the intervention attempt was unsuccessful in the cath lab resulting in referral for surgery and investigations where RSOV was caused by previous interventions or endocarditis were excluded.

### Data collection

The search results were screened based on the relevance of their abstracts and titles. The full text of the selected studies was obtained. Two independent authors (AA and NT) further assessed the included studies based on the inclusion criteria. The reviewers consulted a senior author (AH) to achieve a consensus in the event of differing opinions. The studies that seemed to report overlapping cases were excluded. The selected articles are sorted in Table 1 based on study type and year. The authors identified 15 main variables from the final articles: the name of the author, the year of publication, the type of study, the location of the study, the number of studied patients, the study population's mean age, the studied patients' gender, defect sizes and defect sites, occluder device types and sizes, follow-up durations, complications, accompanying lesions, and success rates.

**Table 1:** Characteristics of the studies included in the present study.

#	Author/ Year	*N*	Age (mean, range or SD, y)	Male gender *n* (%)	Site of defect, (*n*)	Defect size median and range or mean ± SD (mm)	QP/QS	Device type, (*n*)	Device size (mm), (*n*)	F/U Duration (mon), median and range or mean ± SD	Complication (*n*)	Associated untreated significant lesions (*n*)	Success rate (%)
1	Yang (11)	26	39 (13–72)	14 (53.8)	RCS–RA, 13 RCS–RV, 6 NCS–RA, 7	7 (3–14)	NR	–PDAO (LifeTech Scientific Corp), 25 –VSDO ( Starway Medical Technology Co), 1	NR	24 (1–84) in 25 patients	AR: 2 Residual shunting: 3 Recurrence of RSOV: 1 Moderate PE: 1	Moderate-to-severe TR: 1	100%
2	Galecza (12)	16	39.9 (15–79)	11 (69)	RCS–RA, 2 RCS–RV, 2 NCS–RA, 12	9.4 (4–16)	1.4–3.7	–ADO, 22 –ASO, 1 –MVSDO, 3	–ADO 8/6, 1 –ADO 14/12, 5 –ADO 10/8, 2 –ADO 12/10, 4 –ADO 16/14, 1 –ADO 22/20, 1 –ASO 6, 1 –MVSO 10, 1	Rnage: 1–132	Unresolved residual shunting: 1 PVC and supraventricular tachycardia:1 Recurrence of RSOV: 3	Coarctation of the aorta BAV with significant AR: 1	100%
3	Samson (13)	24	34.9 ± 12.3	18 (75)	RCS–RA, 4 RCS–RV, 10 RCS-RV, 2 RCC–LV, 1 NCS–RA, 4 NCS–RV, 3	6.3 ± 1.7	NR	–PDAO Cardi-O-Fix, 2 –PDAO, 21 –Vascular Plug II: 8 mm	–PDA Device size: 4/6, 1 6/8, 1 10/8, 3 12/10, 5 14/12, 4 16/14, 5 18/16, 4	8 (median )	Postprocedural LV systolic dysfunction (pulmonary edema): 1 Death: 1 Significant residual shunting: 1 Trivial residual shunting: 5 Pulmonary device embolization: 1	AI: 3	91.70%
4	Yang (14)	29	39 (13–72)	15 (52)	RCS–RA, 12 RCS–RV, 7 NCS–RA, 8 NCS-RV, 1 RCS-RAV, 1	6.4 ± 2.4 Range: (3–14 )	NR	–PDAO (LifeTech Scientific Corp), 28 –VSDO ( Starway Medical Technology Co), 1	4.3 ± 1.7 mm (2–8 mm larger than defect)	30 (1–83)	Trivial AR: 2 Resolved residual shunting: 2 Recurrence of RSOV: 1 (referred for surgery)	VSD: 2 BAV: 1 Moderate MR: 1 Mild PE: 1	100%
5	Xiao (15)	29	36.7 ± 11.1	21 (72 )	RCS–RV, 15 RCS–RA, 7 NCS–RA, 7	Mean: 6 Range (4–10)	1.3–2.8	–VSDO, 18 –PDAO, 11	VSDO: mean = 10 mm PDAO: mean = 14 mm	89.4 ± 34.9	Severe AI: 1 (was excluded) Mild residual shunting: 1	VSDO: 2 AR: 1 PDA: 1	96.70% Counting 1 residual shunt and sever AI All 30 Cases were closed successfully.
6	Awasthy (16)	12	38.3 (21–59)	10 (84)	NCS-RA, 8 NCS-RVOT, 1 RCS-RA, 1 RCS-RVOT, 2		NR	–PDAO (LifeTech), 12	16/14, 9 14/12, 1 12/10, 1 10/8, 1	30 (1–84)	1 had cardiac arrest in an attempted crossing of the RSOV (excluded)		100%
7	Sinha (17)	8	26.1 ± 6.9	5 (60.3)	RCS–RA, 1 RCS–RV, 1 NCS–RA, 6	13 (9–17)	NR	–CDO (Vascular Innovation), 8	20/18, 2 12/10, 2 18/16, 2 16/14, 2	12 (9–26)	Resolved mild residual shunting: 3	BAV: 1 Severe TR: 2 RVD: 2	100%
8	Mahimarangiah (18)	24	29 (14–72)	NR	RCS–RA, 4 RCS–RV, 7 RCS–RVOT, 7 NCS–RA, 3 NCS–RV, 3	Range: 4–16	1.6–3.8	–PDAO, 20 ADO-II: 4/6–6/6 –MVSDO, 4	PDAO: 8/6–20/18 –ADO-II, 2 MVSDO: 7–10	Rnage: 6–54	Residual leakage: 2 Severe AR: 1 Recurrence: 1	Small VSD: 1 *BAV: 3	88%
9	Sinha (19)	7	25 (16–48)	3 (43)	RCS–RA, 1 RCS–RVOT, 3 NCS– RA, 3	8 (6–10)	Mean:2.8	–CDO (Vascular Innovation)	10/8, 2 12/10, 2 14/12, 2	34 (1–55)	Resolved residual shunting: 1	BAV: 1	100%
10	Zhong (20)	22	30.3 (18–48)	15 (68)	RCS–RA, 2 RCS–RVOT, 8 NCS–RA, 11 NCS–RV, 1	Mean: 7 Range: (5–10)	1.3–2.5	–ADO (AGA) ,19 –MVSDO (AGA), 1 The device used for 2 patients resulted in AI (were not reported)	–ADO: 12/10, 9 14/12, 7 16/14, 2 8/6, 1 –MVSD 8, 1	Mean:19 Range: (3–35)	Residual shunting: 2 Severe AR (leading to surgery): 2	BAV: 1 AVR: 1	91%
11	Fang (21)	16	33 (4–58)	8 (50)	RCS–RA, 3 RCS–RV, 9 NCS–RA, 1 NCS–RV, 3	7.5 (4–13)	1.1–3.0	–PDAO	8/6, 1 10/8, 7 12/10, 4 14/12, 2 18/16, 1	Rnage: 18–102	Incomplete RBBB: 1 Hemolysis (due to small residual shunting leading to repeat device closure): 1 1st degree AVB: 1	VSD: 1	100%
12	Tong (22)	13	31.1 (18–38)	9 (69)	RCS–RA, 3 RCS–RV, 5 NCS–RA, 1 NCS–RV, 3 LCS–RVOT, 1	9.2 (6–12)	NR	–DO (AGA or Starway)	12/10, 2 16/14, 4 10/8, 3 14/12, 4	43 (12–60)	Resolved small residual shunting: 4	None	100%
14	Liu (23)	24	45 (24–74)	15 (63)	RCS-RA, 7 NCS-RA, 7 RCS-RV, 7 NCS-RV, 3	5.4 (4–8)	1.6–4*	SWDO, 17 MDO, 5 Asymmetric, 2	–SWDO A4B2, 14 –SWDO A6B2, 3	19 (6–96)	Hemolysis: 2 Trivial residual shunting: 3 Mild AI: 5 at discharge and just 1 mild AI at follow-up	VSD: 3 Significant AR: 1	92%
15	Guan (24)	10	40 (19–63)	6 (60)	RCS–RA, 2 RCS–RV, 6 NCS–RA, 2	10.1 (7–15)	1.4–3.1	PDAO, 8 VSDO, 2	–PDAO 2–4 mm larger than defect –VSDO 3–5 mm larger than defect	Range: 13–48	AR: 1 Myocardial infarction: 1	Small subaortic VSDO: 2 Previous IE: 1 VSD: 2	100%
16	Li (25)	4	46.2 (40–59)	3 (75)	RCS–RA, 2 RCS–RVOT, 1 RCS->RV, 1	7.8 (6–11)	NR	PDAO (Starway Medical Technology Inc)	10–17	Rnage: 8–30	Resolved mild residual shunting: 1	None	100%
17	Kerkar (26)	18	27 (21–52)	10 (56)	RCS–RA, 4 RCS–RVOT, 5 NCS–RA, 8 NCS–RV, 1	9 (4–11)	1.5–3.2	ADO (AGA)	16/14, 6 14/12, 4 12/10, 4 10/8, 2 8/6, 4	24 (1–60)	Small residual shunting: 4 (resolved in 2 patients) Resolved moderate residual shunting: 1 Trivial AI: 4 (resolved in 2) Hemolysis: 1	BAV: 1 CoA: 1 ASD: 1	100%
18	Sivadasanpillai (27)	7	44.8 (28–62)	4 (57)	RCS–RA, 4 RCS–RV, 1 RCS–RVOT, 1 NCS–RA, 1	8.3 (3–12)	1.5–3.6	NDO	14/12, 2 16/14, 1 18/16, 1 12/10, 1 10/8, 1 6/4, 1	9.3 (8–17)	Resolved trivial residual shunting: 2	BAV 1 Surgical repair recurrence + ventricular failure + AVR: 1 Biventricular failure: 1	100%
19	Sen (28)	8	32.8 ± 10.0	5 (63)	RCS–RA, 2 RCS–RVOT, 2 NCS–RA, 3 NCS–RVOT, 1	NR	NR	PDAO (LifeTech Scientific)	10/8, 2 12/10, 3 14/12, 2	11.3 ± 4.1	Transient sinus bradycardia: 1 AI: 1	None	100%
20	Szkutnik (29)	4	32.2 (23–51)	3 (75)	RCS–RA, 3 RCS–RV, 1 NCS–RA, 1	6 (5–13)	1.7–2.2	ADO, 5 ASO, 1	–ADO: 14/12, 1 10/8, 2 8/6, 2 –ASO: 6	13.6 (9–19)		ToF correction: 1 Surgical closure of RSOV: 1	100%
21	Zhao (30)	10	37 (7–69)	4 (40)	RCS–RV, 5 RCS–RA, 3 NCS–RA, 2	Mean: 6 Range: (2–10)	1.2–2.7	ADO (AGA)	8, 5 10, 2 4, 1 6, 1 12, 1	3	Resolved traces of a residual shunt: 1		100%
22	Chang (31)	4	27 (18–47)	2 (50)	NCSV–RA, 1 RCSV–RA, 1 RCSV–RV, 2	Range: (4–8)	1.4–2.6	ADO, 3 GCO, 1	–ADO 12/10, 1 10/8, 2 –GCO 38-8-5, 1	Rnage: 3–18	Resolved small residual shunting in a patient with GCO	VSDO and AVR due to IE: 1 (excluded)	100%
23	Arora (32)	8	24.1 (14–35)	8 (100)	RCS–RV, 5 NCS–RA, 3	Range: (7–12)	2–3.5	RUD, 2 ASO, 1 ADO, 6	–RUD: 2 –ASO 16, 1 –ADO 14/12, 5 12/10, 1	Rnage: 2–96	Residual shunting, RVOT obstruction and persistent hemolysis referred for surgery: 1 1 died 6 months after progressive CHF	None	87.50%
24	Mumtaz (33)	12	38 ± 15	8 (67)	RCS-RVOT, 9 RCS or NCS-RV, 1 NCS-RA, 3	10 ± 3	NR	DO	Initial device size 10/8, 4 12/10, 1 14/12, 1 16/14, 3 18/16, 3 In 3 patients the upsize device was selected.	52 (1–75)	IE: 1 VT (ablation leading to complete heart block and pacemaker implantation): 1	VSD: 12	100%
25	Case Reports (34–104)	72	30.8 ± 14.3	53 (76.5%)	NCS-RV, 3 NCS-RA, 27 RCS-RV, 26 RCS-RA, 10 RCS-LV, 1 LCS-LV, 1 LCS-RA ,1 LPS-RV, 1 NCS-AVC, 1 NR, 1	Range: (3–18)	NR	ADO, 28 PDA, 15 ASO, 2 VSDO, 14 PFOO, 3 CDO, 3 RU, 1 VP, 4 Coil, 1 DDO, 1	2–25	6.1 ± 9	Mild shunting: 19 Significant shunting: 3 Device emboli: 3 Mild AI: 1 Significant AI: 3 RVOT obstruction: 1 Thrombus: 1 Transient block: 2 Transient LV dysfunction: 1	VSD: 4 ASD: 3 AVR: 3 BAV: 2 CoA: 2 DORV: 1 PFO: 1 Previous RSV: 1 Sub Aortic membrane: 1 PDA: 1 Coronary cameral Fistula: 1 TGA: 1	93%

ADO, amplatzer duct occluder; AI, aortic insufficiency; AR, aortic regurgitation; ASD, atrial septal defect; ASO, atrial septal occluder; AV, atrioventricular; AVP, amplatzer vascular plug; AVR, aortic valve replacement; BAV, bicuspid aortic valve; CDO, cardifix duct occluder; CHF, congestive heart failure; CoA, coarctation of the aorta; DORV, double-outlet right ventricle; EI, infective endocarditis; GCO, gore cardioform septal occluder; LA, left atrium; LCA, left coronary artery; LCS, left coronary sinus; MDO, muscular duct occluder; MR, mitral regurgitation; MVSDO, muscular ventricular septal defect occluder; NCS, non-coronary sinus; NDO, nit duct occluder; NR, not reported; PA, pulmonary artery; PDAO, patent ductus arteriosus occluder; PE, pleural effusion; PFOO, patent foramen ovale occluder; RA, right atrium; RBBB, right bundle branch block; RCS, right coronary sinus; RSOV, ruptured sinus of valsalva; RUD, rashkind umbrella device; RV, right ventricle; RVD, right ventricular dysfunction; RVOT, right ventricular outflow tract; SWDO, small waist double-duct occluder; TGA, transposition of the great arteries; ToF, tetralogy of Fallot; TR, tricuspid regurgitation; VSDO, ventricular septal defect occluder.

### Search results

**Figure 1:**
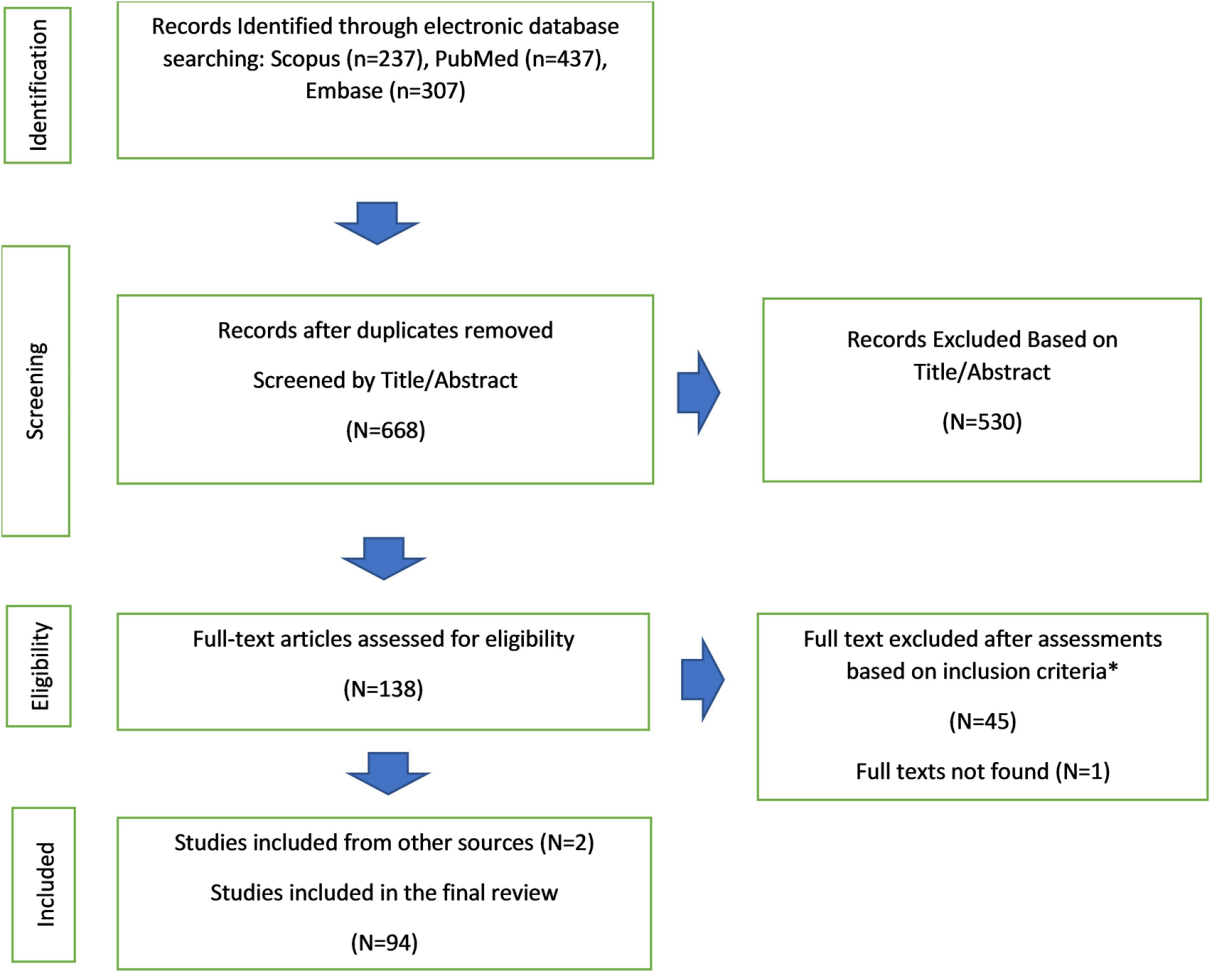
The image depicts the present study's systematic review PRISMA flow diagram describing database searches, title/abstracts screenings, and full-text assessments.

A report of the search results based on the PRISMA guideline is presented in Figure 1. Our search in the 3 databases yielded 437, 273, and 307 articles from PubMed, Scopus, and Embase, respectively. The removal of duplicates left 668 articles. Another 530 studies were excluded based on our title and abstract relevance criterion. Afterward, the full texts of the remaining 138 articles were obtained, and their reference lists were searched for further related studies, leading to the addition of 2 new articles. Despite multiple attempts to contact both the author and the journal, obtaining the full text of 1 article proved impossible. Then, full-text reviews of the selected articles excluded another 46 investigations based on our inclusion criteria (Supplementary Table S2).

Ultimately, the current review was conducted on 94 articles: 23 case series and 71 case reports. The included articles are summarized in Table 1, with the case report combined in the last row. A complete list of the included case reports is available in (Supplementary Table S3).

## Results

The current study reviewed 94 articles presenting 407 cases, 377 adults, and 30 pediatric patients (<18 y/o). Males accounted for about 65% of the studied patients, with an average age of 34.5 years.

A summary of the patients' symptoms is presented in Table 2. Totally, 368 patients (90.2%) reported symptoms, the most prevalent of which were dyspnea [*n* = 150 (37.3%)], chest pain [*n* = 65 (16%)], palpitations [*n* = 65 (16%)], lower extremity edema [*n* = 50 (12.3%)], and fatigue [*n* = 20 (4.9%)]. Ruptures were unexpectedly discovered during imaging in 36 asymptomatic individuals (8.8%). Records of the New York Heart Association functional class were available on 311 patients: class I in 44 patients (10.8%), class II in 115 (28.3%), class III in 111 (27.3%), and class IV in 41 (10.1%).

**Table 2:** Symptoms of patients with ruptured sinus of valsalva undergoing transcatheter closure.

Symptom	Frequency (%) (*N* = 407)
Dyspnea	152 (37.3%)
Chest pain	65 (16%)
Palpitations	65 (16%)
Lower extremity edema	50 (12.3%)
Fatigue	20 (4.9%)
Orthopnea	5 (1.2%)
Syncope	5 (1.2%)
Dizziness	3 (0.7%)
Coughs	3 (0.7%)
Headaches	1 (0.2%)
Vomiting	1 (0.2%)
Abdominal pain	1 (0.2%)
Diarrhea	1 (0.2%)
White-foamy sputum	1 (0.2%)
Excessive sweating	1 (0.2%)
Failure to Thrive	1 (0.2%)
Recurrent respiratory infection	1 (0.2%)
Hypotension	1 (0.2%)
Asymptomatic	36 (8.8%)
NR	36 (8.8%)
New York Heart Association Classification	
I	44 (10.8%)
II	115 (28.3%)
III	111 (27.3%)
IV	41 (10.1%)
Not Reported	96 (23.6%)

**Figure 2:**
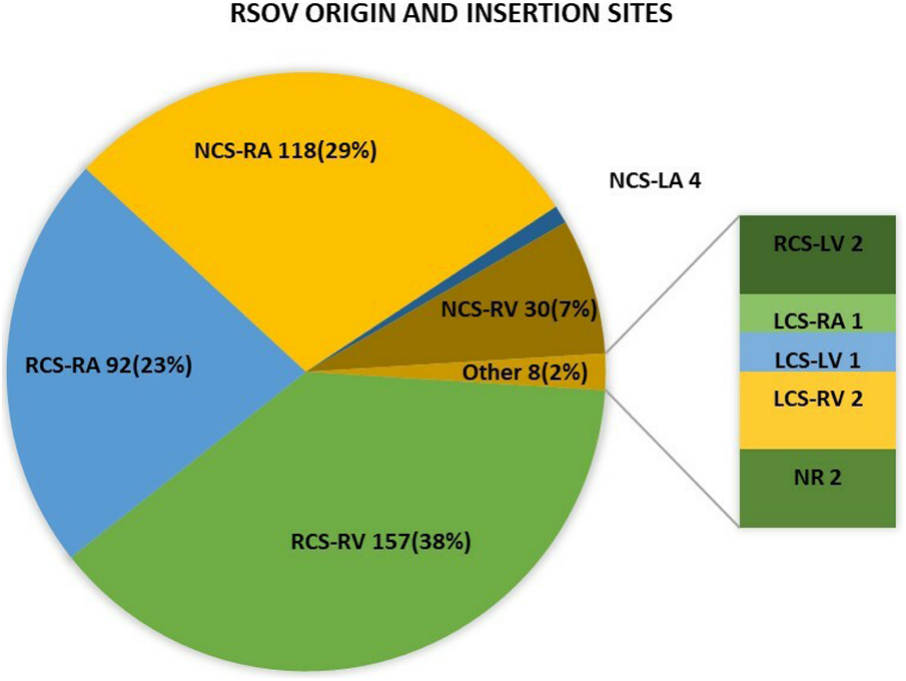
The image illustrates the prevalence of the origin and insertion sites of the ruptured fistula in RSOV patients who underwent TCC. RSOV, ruptured sinus of valsalva; TCC, Transcatheter closure; RCS, (right coronary sinus; LCS, left coronary sinus; NCS, non-coronary sinus; RA, right atrium; RV, right ventricle; LA, left atrium; PA, pulmonary artery; NR, not reported.

The origin and insertion sites of the RSOV fistula are depicted in Figure 2. Most ruptures [*n* = 251 (61.7%)] originated from the right coronary sinus, and 152 ruptures (37.3%) originated from the non-coronary sinus. The left coronary sinus was rarely the origin [*n* = 4 (1.0%)]. A wide range of defect sizes was reported in the literature, from 3 mm to 17 mm (aortic opening size). The most common opening site of ruptures was the right atrium [*n* = 211 (51.8%)], followed by the right ventricle [*n* = 189 (46.4%)].

The selection criteria for TCC were reported in 256 cases. The most common criterion reported for 77% of the cases was the lack of associated cardiac anomalies (other than RSOV) requiring surgery. All the studied patients underwent the TCC of RSOV with a total success rate of 95.6%. Multiple device types were utilized for RSOV closure (Table 3). Patent ductus arteriosus (PDA) occluders were the devices most frequently utilized for the intervention [*n* = 172 (41.7%)], followed by the Amplatzer Ductal Occluder [*n* = 85 (20.6%)]. Device size was based on the size of the defect assessed by transesophageal echocardiography (TEE) or other imaging modalities.

**Table 3:** Frequency and prevalence of device types utilized in the transcatheter closure of ruptured sinus of valsalva.

Device	Frequency (%) (*N* = 407)
Patent Ductus Arteriosus Occluder	172 (41.7%)
Amplatzer Duct Occluder	85 (20.6%)
Other Duct Occluders	43 (10.4%)
VSD Occluder	36 (8.7%)
Cardifix Duct Occluder	18 (4.4%)
Small Waist Double-Duct Occluder	17 (4.1%)
Muscular VSD Occluder	11 (2.7%)
Nit Duct Occluder	7 (1.7%)
Atrial Septal Occluder	5 (1.2%)
Vascular Plug	5 (1.2%)
Rashkind Umbrella Device	3 (0.7%)
Patent Foramen Ovale Occluder	3 (0.7%)
ASYMMETRIC DO	2 (0.5%)
Gore Cardioform Septal Occluder	1 (0.2%)
Modified double-disk ventricular occluder	1 (0.2%)
Coil	1 (0.2%)
Not Reported	2 (0.5%)

Significant complications were reported in 49 patients (12%) (Table 4). Residual shunts were reported in 61 patients (15%); still, they were resolved in most cases, with only 6 patients having significant remaining shunts. A new onset or progression of aortic insufficiency, followed by device embolization, was reported in 22 patients (5.4%); nevertheless, only 7 patients (1.7%) had moderate or severe insufficiency. Other notable complications were recurrence [*n* = 6 (1.5%)], hemolysis [*n* = 5 (1.2%)], and device embolization [*n* = 4 (1%)]. Post-intervention mortality was reported in only 2 patients.

**Table 4:** Prevalence of reported complications in the transcatheter closure of ruptured sinus of valsalva.

Complication	Frequency (%) (*N* = 407)
Resolved residual shunts	55 (13.5%)
Mild AI	15 (3.7%)
Significant AI	7 (1.7%)
Significant residual shunts	6 (1.5%)
Recurrence of RSOV	6 (1.5%)
Hemolysis	5 (1.2%)
Device embolization	4 (1%)
RVOT obstruction	2 (0.5%)
Pulmonary effusion	2 (0.5%)
Transient atrioventricular block	2 (0.5%)
LV dysfunction	2 (0.5%)
Death	2 (0.5%)
Heart block	3 (0.7%)
Thrombosis	1 (0.2%)
Premature Ventricular Contractions	1 (0.2%)
Left Coronary Artery compression	1 (0.2%)
Femoral Arteriovenous fistula	1 (0.2%)
Right bundle branch block	1 (0.2%)
Infective endocarditis	1 (0.2%)
Myocardial infarction	1 (0.2%)
ST depression	1 (0.2%)
RCC prolapse	1 (0.2%)

RSOV, Ruptured Sinus of Valsalva; AI, Aortic Insufficiency; LV, Left Ventricle; RVOT, Right Ventricular Outflow Tract; RCC, Right Coronary Cusp.

## Discussion

Performing the TCC of RSOV rather than surgical treatment has been dramatically favored since its introduction in 1994. However, a comprehensive guideline on the indications, patient selection, choice of imaging modalities, device selection, and complication prevention is still lacking.

Our current review of the literature on TCC yielded 407 patients undergoing the TCC of RSOV at a mean age of 34.5 years old. The most reported symptoms were dyspnea, chest pain, and palpitations. Most SOVA cases originated from the right coronary sinus, and the right atrium was the most common rupture insertion site. PDA occluders were utilized for almost one-third of the studied patients. Complications were reported in only 12% of the cases, with aortic insufficiency and residual shunting accounting for the majority of these complications.

### Signs and symptoms

Patients with unruptured SOVA are often symptom-free, whereas a rupture into a heart chamber can significantly change hemodynamics and create severe symptoms (15). Dyspnea, palpitations, chest pain, fatigue, and peripheral edema are the most frequently reported symptoms after SOVA rupture. The onset of these symptoms can be acute or gradual (3, 101).

The pressure difference between the aorta and the low-pressure heart chamber causes continuous machinery murmurs, frequently heard in patients with RSOV. The murmurs become more intense when a fistula grows more prominent (102).

### TCC vs. Surgical Closure

Since the first reported case of the TCC of RSOV in 1994, mounting evidence (mostly case reports and a small number of case series) has indicated the effectiveness of TCC as a potential alternative to surgical intervention. TEE proves its effectiveness during the intervention by providing live visualization of cardiac structures, especially the aortic valve. Multiple imaging modalities have made percutaneous approaches a more feasible treatment option than open-heart surgery. Still, surgical intervention is unavoidable in RSOV cases with accompanying heart defects, infections, arrhythmias, or outflow tract obstruction. Moreover, prompt surgery might become necessary when TCC results in major complications, such as significant residual shunts, aortic insufficiency, and device embolization.

### Patient selection for TCC

Precise patient selection criteria are critical before TCC. Xiao et al. (15) considered patients to have TCC if they have a body weight exceeding 10 kg, if the non-coronary sinus or the right coronary sinus is the origin of the defect rupturing into the right ventricle or the right atrium, if the defect size is less than 10 mm, if RSOV does not affect the aortic valve and has more than a 7 mm distance from the annulus of the aortic valve, if a gap of more than 5 mm exists between the ostium of the right coronary sinus and the ruptured site, and if surgery is needed in the absence of other cardiac anomalies.

Lui et al. (22) also suggested that RSOV patients with a European System for Cardiac Operative Risk Evaluation II (EuroSCORE II) score exceeding 20% would benefit from TCC. Nevertheless, controversy remains over the indications for TCC in patients with RSOV since this method is new and the prevalence of RSOV is low. Further studies are, therefore, required to validate the indications or contraindications of TCC in patients with RSOV (11).

### Imaging

Although there are no stringent guidelines on RSOV imaging modalities, recent studies have proposed a multimodality imaging approach to RSOV (103, 104).

Transthoracic echocardiography (TTE) is the first-line modality for diagnosing RSOV (104). A primary TTE examination can detect aortic root dilation at the level of the Valsalva sinus (7) and assess the RSOV dimension.

Color Doppler echocardiography can visualize the blood flow through the RSOV fistula from the aorta into a heart chamber (105).

TEE provides higher-resolution images due to its proximity to the heart and the thoracic aorta. During corrective interventions, TEE can visualize RSOV (106). Further, the relative accuracy of TEE in defect sizing has reduced the need for balloon occlusive diameter methods during TCC (107).

Multidetector computed tomography and cardiac magnetic resonance imaging (CMR) can assess the aorta fully by forming a 3D reconstruction. Multidetector computed tomography or CMR are also capable of determining the dimensions, morphology, and complications of RSOV.

Electrocardiogram-gated CT or CMR can augment imaging accuracy by controlling motion artifacts (108, 109).

Invasive transcatheter angiography is occasionally necessary to differentiate RSOV from other coronary disorders (7).

### Transcatheter occluder devices

The first case of the TCC of RSOV was performed utilizing a Rashkind Umbrella via an arterial approach (110). However, the transvenous approach has been performed more frequently because of its higher maneuverability and easier access to the Valsalva sinus. Recent decades have seen the use of other devices, including Amplatzer ductal occluders, PDA occluders, muscular ventricular septal defect occluders, septal occluders, and coils, depending on the anatomy of the defect.

Amplatzer-type ductal occluder devices have been in frequent use due to the conformity of their shape with RSOV morphology (20). Some studies have reported a preference for PDA occluder use. Other than the characteristics of the defect, the interventionist's familiarity with the device, the availability of the device, and financial concerns are taken into consideration in device selection (111).

### Summary of the procedure

TCC involves creating an arteriovenous loop by passing a Judkins right coronary catheter from the ascending aorta through the defect into the right atrium or the right ventricle with the aid of an angled-tip guidewire (25, 110). The guidewire is, then, exchanged for a long guidewire, and a gooseneck snare is introduced through venous access to snare the long guidewire in the right atrium or the pulmonary artery with a view to avoiding entrapment and damage to the chordae tendineae of the tricuspid valve, which could result in postprocedural tricuspid regurgitation. Subsequently, a long wire is exteriorized from venous access to form a stable arteriovenous loop crossing the RSOV. Thereafter, a delivery sheath is introduced through the femoral vein and is negotiated from the RSOV to the ascending aorta while precaution is taken to avoid damage to the surrounding tissues, causing heart blocks or defect dilation (111). In the next step, an appropriate-sized device is selected and connected to a delivery cable. The device is inserted through this sheath, and the aortic disk is opened in the aorta. Next, the delivery sheath and the delivery cable are pulled back so that the disk closes the aortic side of the RSOV. After the confirmation of the appropriate placement, the other disk is deployed in the associated right chamber (110, 112).

It is essential to assess all the steps of the procedure with intraprocedural TEE for the confirmation of the complete blockade of the shunt. Possible residual shunting, aortic regurgitation, tricuspid regurgitation, and other possible procedural complications should also be assessed following complete device emplacement.

### Complications

Although shown to be effective, the TCC of RSOV can be associated with severe complications on rare occasions. Possible complications can be prevented or readily addressed through efficient measures. A summary of reported complications is presented in Table 4.

The new onset or progression of an existing aortic regurgitation is the most critical complication in the TCC of RSOV. Aortic insufficiency can be due to traction on the aortic valve annulus or an aortic valve injury during the procedure. In cases of severe aortic insufficiency, surgical intervention is unavoidable.

Compression of the coronary arteries after device insertion can also be a life-threatening complication, which can be avoided by visualizing coronary artery flow during the intervention via angiography. A new-onset ST depression after device implantation can also be due to coronary artery compression. If left undetected, the condition could cause myocardial infarction (109). Embolization of the occluder device can also be a possible complication. Glaceza et al. (109) reported the embolization of undersized devices in 3 cases; they were, however, able to retrieve the migrated devices percutaneously.

Hemolysis is also a rare complication of TCC caused by the inserted device (46).

In rare situations, disturbances in the conduction pathways might result in arrhythmias, including atrioventricular or bundle branch blocks. These blocks are caused by possible traction or compressions on conductive pathways. Later inflammations could also be a cause of these arrhythmias (112).

As with any percutaneous intervention, complications associated with catheterization sites, including atrioventricular fistulae, hematoma, and bleeding events, are also possible.

### Limitations

The lack of large-scale studies and clinical trials was the principal limitation of the present study.

## Conclusions

RSOV is a rare pathology, and scarce data are available in the literature. The TCC of RSOV is a practical approach with acceptable safety and a high success rate. Most of the data reviewed in the present study were based on expert opinion, case reports, and case series. Hence, large-scale clinical trials/cohorts with extended follow-up periods are needed for elucidation.
