# Supplementary material for: Transcatheter closure of a ruptured sinus of valsalva: a systematic review of the literature
Source: Front Cardiovasc Med. 2023 Aug 25;10:1227761. doi: 10.3389/fcvm.2023.1227761 (PMC10505828; doi:10.3389/fcvm.2023.1227761)
Supplement: Supplementary file 1 [file Datasheet1.docx]

| **Table S1:** Search syntax and results from the 3 databases searched in the present study | | | |
| --- | --- | --- | --- |
| **Date** | **Database** | **Search Syntax** | **Number of Results** |
| 12. Jul.2022 | PubMed | All Fields: (Valsalva) AND (Sinus) AND (Rupture) OR (Aneurysm) AND (transcatheter) OR (catheter) OR (Device) | 437 |
| 12. Jul.2022 | Scopus | (TITLE-ABS-KEY (“Valsalva” AND “Sinus”) AND (TITLE-ABS-KEY (“Rupture” OR “Aneurysm”) AND (TITLE-ABS-KEY (“Transcatheter” OR “catheter” OR “Device”) | 273 |
| 12. Jul.2022 | Embase | Quick search: All Fields: (“Valsalva” AND “Sinus”) AND (“Rupture” OR “Aneurysm”) AND (“transcatheter” OR “catheter” OR “Device”) | 307 |

| **Table S2:** Articles excluded from the present study after a full-text review | | | |
| --- | --- | --- | --- |
| **Title** | **Year** | **Authors** | **Exclusion Reason** |
| Valsalva sinus perforation into the right atrium due to infective endocarditis of transcatheter heart valve | 2015 | Naganuma, T. and Takagi, K. and Fujino, Y. and Kobayashi, T. and Mitomo, S. and Akita, M. and Nakao, T. and Nakamura, S. | Open Surgery |
| Percutaneous transcatheter closure of ruptured sinus of valsalva aneurysm Immediate result and long-term follow-up | 2014 | Sinha, S.C. and Sujatha, V. and Mahapatro, A.K. | Repeated Cases |
| Transcatheter Closure of Ruptured Sinus of Valsalva Aneurysms With An Amplatzer Occluder | 2009 | Szkutnik, M. and Kusa, J. and Glowacki, J. and Fiszer, R. and Bialkowski, J. | Repeated Cases |
| Transcatheter closure of a ruptured valsalva sinus aneurysm using domestic ventricular septal defect occluder | 2007 | Hong, L. and Wang, H. and Li, L.-F. and Cheng, Z.-Q. and Lai, X.-L. and Yin, Q.-L. and Qiu, Y. and Li, B. | Repeated Cases |
| Follow-up of patients with interventional closure of ventricular septal defects with Amplatzer Duct Occluder II. | 2015 | Kanaan M and Ewert P and Berger F and Assa S and Schubert S | Only VSD |
| Double helical suture for transaortic closure of the sinus of Valsalva aneurysm. | 2018 | Fulquet-Carreras E and Di Stefano S and Ortega C and FernÃ¡ndez M | Open Surgery |
| Percutaneous treatment of an iatrogenic pseudoaneurysm of the aortic Valsalva sinus. | 2018 | Montone RA and Bedogni F and De Marco F | Not Related to Coronary Aneurysms |
| Percutaneous left ventricular outflow tract pseudoaneurysm repair | 2021 | Piryanka, K. and Reimer, L. and Berarducci, L. | LVOT Pseudoaneurysms |
| TEE guidance of percutaneous closure the inlet and exit of ascending aorta pseudoaneurysm in a patient had triple valve replacement | 2020 | Abdo Naeim, H. and Alharbi, I.H. and Alamoudi, OSAMA and Saeed, W.M. and Abuelatta, R.A. | Pseudoaneurysms |
| Mediumand long-term follow-up of transcatheter closure of ruptured sinus of Valsalva aneurysm Polish and Ukrainian experience | 2019 | Yashchuk, N. and Szulik, D. and SmerdziÅ„ski, S. and Lazoryshynets, V. and Fiszer, R. and BiaÅ‚kowski, J. and GaÅ‚eczka, M. and Ditkivskyy, I. and Szkutnik, M. and Cherpak, B. and Knop, M. | Repeated Cases |
| Amplatzer device closure of a ruptured sinus of Valsalva aneurysm associated with a transcatheter aortic valve implant | 2018 | Malkun Paz, C. and MuÃ±oz VelÃ¡squez, A. and Isaac Cure, O. and Escobar Modesto, J. and Rebolledo Maldonado, C. | Combined With AVR |
| Percutaneous closure of a postoperative subannular aortic aneurysm via a novel transatrial approach | 2016 | Abdelsalam, S. | Pseudoaneurysms |
| Percutaneous device closure of ruptured sinus of valsalva aneurysm Immediate and mid-term follow-up results | 2015 | Varghese, M.J. and Thompson, V.S. and George, O.K. and George, P.V. and Joseph, G. | Repeated Cases |
| Ruptured sinus of Valsalva aneurysm closure with new types PDA devices | 2015 | Szkutnik, M. and Fiszer, R. and Bialkowski, J. | Repeated Cases |
| Transcatheter closure of ruptured sinus of Valsalva aneurysm with new types of nitinol wire mesh PDA occluders-short and midterm results | 2015 | Szkutnik, M. and Fiszer, R. and Bialkowski, J. | Repeated Cases |
| Transcatheter device closure of ruptured sinus of valsalva Not addressing the pathology, does it make a difference | 2013 | Radhakrishnan, S. and Awasthy, N. | Repeated Cases |
| Transcatheter device closure of ruptured sinus of valsalva Have we achieved the desired objective? | 2013 | Awasthy, N. and Radhakrishnan, S. and Shrivastava, S. and Tomar, M. | Repeated Cases |
| Transcatheter closure of ruptured sinus of valsalva aneurysm with nitinol mesh occluders | 2012 | Bialkowski, J. and Szkutnik, M. | Repeated Cases |
| Transcatheter closure of ruptured sinus of Valsalva aneurysm with nitinol mesh occluders | 2012 | Szkutnik, M. and Fiszer, R. and Bialkowski, J. | Repeated Cases |
| Transcatheter closure of ruptured sinus of valsalva aneurysm with nitinol MESH occluders | 2012 | Fiszer, R. and Szkutnik, M. and Bialkowski, J. | Repeated Cases |
| Percutaneous closure of ruptured sinus of valsalva aneurysm Case series | 2010 | Roshan, J. and George, O. and George, P. and Joseph, G. and Thompson, V. and Chandy, S. | Repeated Cases |
| Transcatheter closure of ruptured sinus of Valsalva aneurysm using the Amplatzer duct occluder Immediate and intermediate-term results | 2010 | Kerkar, P.G. and Nyayadhish, P.Y. and Lanjewar, C.P. and Mishra, N. and Phadke, M.S. and Verma, G.S. and Sharma, A.B. and Mammen, I. | Repeated Cases |
| oSuccessful transcatheter closure of ruptured sinus of Valsalva aneurysm | 2002 | Trehan, VK and Mukhopadhyay, S. and UmaMahesh, C.R. and Yusuf, J. and Arora, R. | Repeated Cases |
| Percutaneous closure of complex paravalvular aortic root pseudoaneurysm and aorta-cavitary fistulas | 2014 | Al-Maskari, S. and Panduranga, P. and Al-Farqani, A. and Thomas, E. and Velliath, J. | Pseudoaneurysms |
| Stabilized Sinus of Valsalva Aneurysm After CoreValve Implantation | 2019 | Amano, T. and Naganuma, T. and Nakamura, S. | Combined With AVR |
| Direct percutaneous puncture and embolization of ruptured sinus of valsalva aneurysm | 2014 | Briggs, J. H. and Tapping, C. R. and Little, M. W. and Uberoi, R. | Open Surgery |
| Transthoracic minimally invasive closure for the treatment of ruptured sinus of Valsalva Aneurysm A case report | 2014 | Cao, X. and Zhang, F. and Wang, L. and Jing, H. and Li, N. | Minimally Invasive |
| Congenital coronary artery fistulas in adults Concomitant pathologies and treatment | 2008 | Cebi, N., Schulze-Waltrup, N., Frömke | Coronary Fistula |
| Delineation of anatomy of the ruptured sinus of valsalva with three-dimensional echocardiography The advantage of the added dimension | 2012 | Chandra, S. and Vijay, S. K. and Dwivedi, S. K. and Saran, R. K. | Endocarditis |
| Catheter inside the right heart for 22 years To intervene or not to intervene? | 2015 | Tolga Çimen,Mehmet Doğan,Ahmet Akyel,Ekrem Yeter | No RSOV |
| Coexisting ventricular septal defect affects the features of ruptured sinus of Valsalva aneurysms | 2017 | Jin, Y. and Han, X. M. and Wang, H. S. and Wang, Z. W. and Fang, M. H. and Yu, Y. and Fang, X. X. | Open Surgery |
| Aorta-right atrial tunnel | 2010 | Krishna, C. S. and Baruah, D. K. and Reddy, G. V. and Panigrahi, N. K. and Suman, K. and Kumar, P. V. N. | No Intervention |
| Peratrial Device Closure of Ruptured Sinus of Valsalva Aneurysm Into Right Atrium | 2019 | Liang, F. and Hongxin, L. and Wenbin, G. and Zhang, H. Z. and Changwe, G. J. | Surgery |
| Off-label use of Amplatzer Duct Occluder II additional sizes | 2017 | Mahmoud, H. T. and Santoro, G. and Capogrosso, C. and Russo, M. G. | Due to Iatrogenic Complications |
| Three-dimensional versus two-dimensional transesophageal echocardiography for device closure of ruptured valsalva sinus aneurysm | 2015 | Mohsenibadalabadi, R. and Hosseinsabet, A. | Letter (No Cases Reported) |
| Successful treatment by percutaneous stent deployment of severe retrograde dissection of the right coronary artery extending into the sinus of Valsalva and ascending aorta [5] | 2005 | Notaristefano, S. and Giombolini, C. and Santucci, S. and Fortunati, F. and Savino, K. and Notaristefano, A. and Ambrosio, G. | No RSOV |
| Successful implantation of HeartWare HVAD left ventricular assist device with concomitant ascending and sinus of valsalva aneurysms repair | 2012 | Takeda, K. and Ahmad, U. and Malaisrie, S. C. and Lee, R. and McCarthy, P. M. and McGee Jr, E. C. | Open Surgery |
| Direct en face imaging of secundum atrial septal defects by velocity-encoded cardiovascular magnetic resonance in patients evaluated for possible transcatheter closure | 2008 | Thomson, L. E. and Crowley, A. L. and Heitner, J. F. and Cawley, P. J. and Weinsaft, J. W. and Kim, H. W. and Parker, M. and Judd, R. M. and Harrison, J. K. and Kim, R. J. | No RSOV |
| Transthoracic minimally invasive closure for the treatment of ruptured sinus of Valsalva aneurysm immediate and mid-term follow-up results | 2022 | Wang, S. and Liu, D. and Li, Y. and Wu, S. and Wang, W. and Ma, Q. and Li, Y. and Wang, W. and Gao, B. | Surgery |
| Ruptured sinus of Valsalva aneurysm in adult age; Percutaneous closure with occluder device. Report of three successful cases | 2015 | Lucelli Yáñez-Gutiérrez, Diana López-Gallegos, Carmen Emma Cerrud-Sánchez,  Horacio Márquez-González, Marlenne Berenice García-Pacheco,  Moisés Jiménez-Santos, Jaime Alfonso Santiago-Hernández,  Key words: Homero Alberto Ramírez-Reyes, Carlos Riera-Kinkel | Not English |
| Intra-aortic balloon clamp for safe resternotomy in a patient with a giant aneurysm of sinuses of valsalva | 2014 | Zembala, M. O. and Filipiak, K. and Niklewski, T. and Przybylski, R. | Open Surgery |
| Iatrogenic left main-stem dissection extending to the circumflex artery and retrogradely involving the left and non-coronary sinuses of Valsalva Iatrogenic aortocoronary dissection | 2015 | Zwolihski, R. and Marcinkiewicz, A. and Jaszewski, R. and Szymczyk, K. and Pietruszyhski, R. | No RSOV, Dissection |
| Sinus of Valsalva rupture into left ventricular myocardium: presentation and interventional catheterization treatment | 2019 | Latson LA, Gongora E, Adewale OO, Roth T | Pseudoaneurysms After MVR Surgery |
| Percutaneous closure of acute aorto‐right ventricular fistula following transcatheter bicuspid aortic valve replacement. Catheterization and Cardiovascular Interventions | 2017 | Nakamura K, Passeri JJ, Inglessis‐Azuaje I | Aorto-Right Ventricular Fistula Following Transcatheter Bicuspid Aortic Valve Replacement |
| Percutaneous closure of ruptured sinus of Valsalva aneurysm: results from a multicentre experience | 2014 | Suxuan Liu, Xudong Xu, Xianxian Zhao,Feng Chen1,Yuan Bai,Weiping Li, Yigang Zhang ,Cheng Wang ,Jun Xiang ,Guangwei Wu , Xiaoli Chen,  Yongwen Qin | Repeated Cases |

| **Table S3:** Characteristics of the case report studies included in the present review | | | | | | | | | | | |
| --- | --- | --- | --- | --- | --- | --- | --- | --- | --- | --- | --- |
| **Author/ Year** | **Number of Patients** | **Age (y)** | **Gender** | **Site of Defect** | **Defect Size (mm)** | **Occluder Device** | **Device Size (mm)** | **QP/QS** | **Accompanying Lesion** | **Follow-up Duration (mon)** | **Complication** |
| Abidin, 2005 | 1 | 36 | M | NCS-RA | NR | ASO | 14 | NR | PFO | None | Mild residual shunting |
| Agrawal, 2015 | 1 | 25 | F | RCS-RVOT | 8 | ADO-I | 12/14 | NR | VSD, Mild AI | None |  |
| Ahmed, 2015 | 1 | 18 | F | NCS-RV | 10 | Life tech Scientific  Heart PDA occluder | 10/12 | NR | - | None |  |
| Al-Senaidi, 2016 | 2 | 45  39 | F  M | RCS-RV  RCS-RVOT | 10  NR | Cocoon Duct Occluder  Occlutech® Figulla® PFO Occluder  AMPLATZER™ PFO  Occluder | 12/10  23/25  18 | 3  5.2 | -  - | None  None | Minimal shunting  Mild-to-moderate residual shunting  (Surgery was done a few months later due to significant shunts from other small defects.) |
| Altekin, 2011 | 1 | 26 | F | NCS-RA | 8 | ADO-I | 10/8 | 3.5 | - | 1 |  |
| Altekin, 2013 | 1 | 30 | M | RCS-RV | 8,6 | ADO-I/ADO-I | 10/6 and 10/6 | 3.8 | - | None | Acceptable residual shunting |
| Arı, 2019 | 1 | 36 | M | NCS-RA | 6 | Cone Shape PDA Occluder | 8/10 | 1.55 | - | 6 |  |
| Aroney, 2018 | 1 | 21 | F | RCS-RA | NR | AVP III | 14×8 | 3 | Subaortic membrane | None |  |
| Asmarats, 2022 | 1 | 39 | M | RCS-RVOT | 7 | AMVSD | 12 |  | VSD | 12 | Minimal residual shunting |
| Bialkowski, 2012 | 1 | 38 | M | NCS-RA | 6 | PDA Cardio-O Fix Occluder | 12/10 | 1.9 | - | 4 |  |
| Bijulal, 2009 | 1 | 47 | M | RCS-RVOT | 16 | Blockaid ductal occluder | 16-14 | 2 | AVR, history of surgical RSOV | None |  |
| Capogrosso, 2018 | 1 | 4 | M | RCS-RVOT | 4 | ADO II‑AS™ | 2-5 | 1.6 | DORV | 3 |  |
| Chua, 2014 | 1 | 44 | M | NCS-RA | 8 | VSDO(AGA) | 10 | NR | - | 12 |  |
| Chen, 2013 | 1 | 41 | F | RCS-RA | 4 | modified double-disk ventricular occluder | 6 | NR | - | 12 |  |
| Choudhry, 2015 | 1 | 44 | M | RCS-RVOT | NR | LifeTech PDA closure device | 16/14 | NR | VSD | None | Embolized after 3 days |
| Cui, 2008 | 1 | 33 | M | RCS-RA | NR | ADO | 12/10 | 4.3 | ASD | 9 |  |
| Cullen, 1994 | 1 | 34 | M | RCS-RV | NR | Rashkind  ductal umbrella | 12 | 2 | - | 6 |  |
| Cullen, 2002 | 1 | 40 | F | RCS-RVOT | NR | ASD | 6 | NR | - | None |  |
| Deore, 2022 | 1 | 55 | M | left posterior sinus -RV inlet | 6 | PDA occluder (LifeTech Inc) | 10/8 | NR | BAV, Coarctation of the aorta | 1 | Early mild residual shunting resolved at follow-up |
| Dhawan, 2020 | 1 | 29 | M | NCS-RA | 6 | ADO | 2.5 | NR | - | None |  |
| Dobarro, 2010 | 1 | 39 | M | NCS-RA | NR | ADO |  | NR | - | None | 1 week later severe AI and AVR |
| Fedson, 2003 | 1 | 54 | M | NCS-RA/RV | NR | 2 ADO (AGA Medical Corp) | 8/6, 8/6 | 1.5 | - | 2 |  |
| Gaio, 2006 | 1 | 55 | F | RCS-RA | 6 | ADO | 8/10 | NR | - | midterm | Early mild residual shunting resolved at follow-up |
| Gioia, 2014 | 1 | 43 | M | NCS-RA | 6 | MVSDO (AGA Medical Corp) | 10 | 2.4 | - | 6 | Trivial residual shunting |
| Gong, 2021 | 1 | 3 | M | NCS-RA | 3 | VSDO | 5 | NR | - | 6 | No substantial residual shunting |
| Hajizeinali, 2019 | 1 | 35 | M | NCS-RA | 3 | Occlutech  PDAO | 6/4 | NR | - | 1 |  |
| İlkay, 2014 | 1 | 37 | M | RCS-RV | 6 | ADO-II |  | NR | - | None |  |
| Jayaranganath, 2010 | 1 | 37 | M | RCS-RVOT | 5 | MVSD intracardiac patch occluder (Life Scientific, Co) | 7 | NR | VSD | 4 |  |
| Jenab, 2021 | 1 | 50 | M | RCS-RA | 8 | Occlutech PDAO | 15/12 | 2.3 | - | None | Mild residual shunt |
| Jiang, 2022 | 1 | 14 | F | NCS-RA | 8 | ADO | 12 |  | - | None |  |
| Karlekar, 2012 | 1 | 40 | M | NCS-RA | 7 | ADO | 10/8 | 3 | - | 6 | Transient CHB |
| Kerkar, 2007 | 1 | 47 | M | NCS-RA | 7 | ADO (AGA Medical Corp) | 14/12 | NR | - | 24 |  |
| Kern, 2020 | 1 | 36 | M | NCS-RA |  | ADO | 8/6 | NR | - | None |  |
| Khan, 2015 | 1 | 27 | M | RCS-RV | 10 | ADO (AGA Medical Corp) | 8/6 | NR | - | 4y |  |
| Khoury, 2010 | 1 | 22 | M | RCS-RV | 8 | ADO (AGA Medical Corp) | 12/10 | 3 | - | 10days | Trivial residual shunting resolved at follow-up |
| Kumar, 2010 | 1 | 34 | M | NCS-RA | 11 | Duct occluder | 14/12 | NR | - | 6 | Transient 1st-degree AVB |
| Kumar, 2017 | 1 | 44 | M | NCS-RA | 8 | PDAO (Life Tech) | 10/8 | NR | - | None | Minimal residual shunting resolved the next day |
| Kumar, 2022 | 1 | 28 | F | NCS-RA | 12 | PDAO(Life Tech) | 16/14 | NR | - | 1 | Transient LV dysfunction |
| Kurşaklıoğlu, 2011 | 1 | 18 | M | RCS-RA | 4 | Occlutech PFOO | 16/18 | 2.1 | - | None |  |
| Mahajan, 2015 | 1 | 34 | M | NCS-RV | NR | PDAO(Life tech) | 12/10 | NR | - | 6 |  |
| Mahimarangariah, 2013 | 1 | 14 | M | RCS-RVOT | 8,? | ADO-I,II | 12/10-6/4 | NR | VSD | None | Mild residual shunting /mild AI |
| Mandel, 2010 | 1 | 28 | M | NCS-RA | NR | Amplatzer MVSDO | 4 | NR | - | 2 | Minimal residual shunting |
| Manuel, 2016 | 1 | 12 | M | RCS-LV | 5 | PDAO (LifeTech) | 10/8 | NR | - | 6 |  |
| Mehta, 2010 | 1 | 37 | F | NCS-RA | 10 | ADO (AGA Medical Corp) |  | NR | ASD | 4 |  |
| Mithani, 2012 | 1 | 10 | F | RCS-RVOT | 10 | Amplatzer VSD device |  | 2 | - | None | Device embolization on the same day |
| Mohan Maddali, 2015 | 1 | 34 | M | RCS-RVOT | 8 | Occlutech  Figulla PFOO, Amplatzer PFOO | 23/25,18 | NR | - | None |  |
| Naeim, 2020 | 1 | 25 | M | NCS-RA | 10 | ADO | 12 | NR | - | 6 |  |
| Narin, 2014 | 1 | 3 | F | RCS-RA | 7 | vascular plug -4 | 4 | 2.9 | - | 2y | Minimal residual shunting |
| Nişli, 2016 | 1 | 10 | NR | NR - RV | 7 | VSDO | 10 | NR | - | 12 |  |
| O H-Icí, 2010 | 1 | 23 | M | NCS-RA | NR | ASO |  | NR | - | None |  |
| Onorato, 2005 | 1 | 48 | M | NCS-RA | NR | ADO | 10/8 | 4 | - | 12 | Device embolization |
| Pahwa, 2015 | 1 | 32 | M | NCS-RA | 11 | LifeTech duct occluder | 18/16 | NR | Coarctation of aorta | 6 |  |
| Palma, 2021 | 1 | 1.5 | M | LCS-RA | NR | ADO-II | 5/4 | NR | - | 12 |  |
| Rao, 2003 | 1 | 9 | M | RCS-RV | NR | long Gianturco coil (Cook) | 10cm | 2.1 | bicuspid  aortic valve, Coarctation of the aorta | 6 | A trivial residual shunting resolved later |
| Rittger, 2015 | 1 | 52 | F | RCS-RVOT | 7 | AVP II | 12 | 3 | - | 3 | Minimal residual shunting |
| Sabiniewicz, 2015 | 1 | 35 | F | NCS-RA | 7 | ADO (AGA) | 6/4 | NR | - | 2y | 2 years later massive thrombus originating at the non-coronary cusp (removed surgically) |
| Sabiniewicz, 2017 | 1 | 46 | M | RCS-RV | 6 | Nit-Occlud Le VSDO | 16/12 | NR | - | 3 |  |
| Santoro, 2007 | 1 | 10 | F | NCS-accessory ventricular chamber | 6, 2 | ADO (AGA) | 8/6 | NR | TA, TGA, Fontan | 18 |  |
| Sarupria, 2012 | 1 | 20 | M | RCA-RA | 6 | ADO | 10/8 | NR | - | None |  |
| Satılmıș, 2013 | 1 | 24 | NR | NCS-RA | NR | PDAO (Cardio Fix) | 14/12 | NR | - |  |  |
| Schaeffler, 2007 | 1 | 11 | NR | RCS-RV | NR | Amplatzer MVSDO | 6 | 1.6 | AVR | 6 |  |
| Shen, 2022 | 1 | 40 | M | NCS-RA | NR | VSDO(Shanghai  Shape Memory Alloy Ltd) | 22 | NR | - | None |  |
| Sinha, 2017 | 1 | 24 | F | RCS-RA | 18 | CDO | 20/18 | NR | - | None | Early mild residual shunting resolved the day after the procedure |
| Song, 2017 | 1 | 63 | M | RCS-RV | NR | AVP/ADO | 16, 10/8 | 2.4 | - | None | Later surgery due to residual shunting |
| Soori, 2021 | 1 | 18 | M | RCS-RVOT | NR | CDO | 18/16 | NR | - | 12 | After 1 year, a residual shunt, moderate AI, and RVOT obstruction (resolved by device surgery) |
| Srivastava, 2012 | 1 | 5 | M | LCS-LV | 8 | ADO (AGA) | 12/10 | NR | PDA, coronary cameral fistula | 36 |  |
| Tang, 2019 | 1 | 45 | M | NCS-RVOT | 7 | Double disk Prem VSDO (LifeTech Scientific) | 10 | 1.6 | AVR | 12 | Minimal residual shunting |
| Vamsidhar, 2015 | 1 | 30 | M | RCS-RVOT | 7 | LifeTech PDAO, Amplatzer MVSDO II | 10/8,10 | NR | - | 12 |  |
| Vatankulu, 2013 | 1 | 29 | M | RCS-RV | NR | ADO | 16/10 | NR | - | None | Trivial residual shunting |
| Wilson, 2015 | 1 | 37 | M | RCS-RA | 6 | Amplatzer MVSDO | 8 | NR | - | 24 |  |
| Zhang, 2015 | 1 | 24 | M | RCS-RVOT | 14 | PDAO | 20/18 | NR | - | 12 | Early mild residual shunting 1 year later (surgery due to AI resulting from the RCS prolapse) |
| ADO (Amplatzer Duct Occluder), AR (Aortic Regurgitation), ASO (Atrial Septal Occluder), AV (Atrioventricular), AVB (Atrioventricular Block), AVP (Amplatzer Vascular Plug), CDO (Cardifix Duct Occluder), CHB (Complete Heart Block), DORV (Double-Outlet Right Ventricle), LA (Left Atrium), LCA (Left Coronary Artery), LCS (Left Coronary Sinus), MDO (Muscular Duct Occluder), MVSDO (Muscular Ventricular Septal Defect Occluder), NCS (Non-Coronary Sinus), NDO (Nit Duct Occluder), NR (Not Reported), PA (Pulmonary Artery), PDAO (Patent Ductus Arteriosus Occluder), PFOO (Patent Foramen Ovale Occluder), RA (Right Atrium), RCS (Right Coronary Sinus), RUD (Rashkind Umbrella Device), RV (Right Ventricle), SWDO (Small Waist Double-Duct Occluder), VSDO (Ventricular Septal Defect Occluder). | | | | | | | | | | | |
